# Complete Genome and Methylome Analysis of the Box-Shaped Halophilic Archaeon Haloarcula sinaiiensis ATCC 33800

**DOI:** 10.1128/MRA.00619-21

**Published:** 2021-08-26

**Authors:** Alexey Fomenkov, Priya DasSarma, Sean P. Kennedy, Richard J. Roberts, Shiladitya DasSarma

**Affiliations:** a Research Department, New England BioLabs, Ipswich, Massachusetts, USA; b Department of Microbiology and Immunology, University of Maryland School of Medicine, Baltimore, Maryland, USA; c Department of Computational Biology, Institut Pasteur, Paris, France; Portland State University

## Abstract

The genome of halophilic archaeon Haloarcula sinaiiensis ATCC 33800 was sequenced and assembled and comprises seven replicons. Four m6A and one m4C modified motifs and their responsible methyltransferase genes have been identified in the genome by single-molecule real-time (SMRT) sequencing and bioinformatic analysis.

## ANNOUNCEMENT

Haloarcula sinaiiensis ATCC 33800 is a halophilic archaeon isolated from brine samples collected at the Red Sea Sabkha Gavish (1). The cells are flat and angular, including irregular rectangles, triangles, and squares. *H. sinaiiensis* is extremely halophilic with a requirement for more than 2 M NaCl for growth and 3 to 4 M NaCl for optimal growth (2–4).

A Pacific Biosciences RS II instrument and SMRTAnalysis pipeline were used for sequencing, contig assembly, and modification analysis as in reference 5. The *H. sinaiiensis* ATCC 33800 strain was a gift from ATCC. Briefly, cells were grown in 500 ml ATCC 1230 *Haloarcula* medium and trace elements (3.50 mg/ml FeSO_4_ · 7H_2_O, 0.88 mg/ml ZnSO_4_ · 7H_2_O, 0.66 mg/ml MnSO_4_ · H_2_O, and 0.02 mg/ml CuSO_4_ · 5H_2_O [pH 7.4]) diluted 2,000-fold at 37°C for 36 h and then centrifuged to recover cells. Pellets were resuspended in 100 ml of a 25% NaCl salt solution and lysed by osmotic shock by addition of a 10 mM EDTA solution until visual clearing occurred. High-molecular-weight DNA was harvested. The archaeal lysate was extracted three times with an equal volume of phenol saturated with Tris-HCl (pH 8) followed by dialysis and treatment with RNase (6).

A total of 10 μg of genomic DNA (gDNA) was used to prepare single-molecule real-time (SMRT) libraries. Briefly, SMRTbell libraries were constructed from a genomic DNA sample sheared to ∼10 to 20 kb using the G-tubes protocol (Covaris, Woburn, MA, USA), end repaired (NEBnext end repair, E6050; New England BioLabs [NEB], Ipswich, MA, USA), and ligated (Quick Ligation kit, M2200S; NEB) to PacBio hairpin adapters. Incompletely formed SMRTbell templates and linear DNAs were digested with a combination of exonuclease III and exonuclease VII (New England BioLabs). DNA qualification and quantification were performed using the Qubit fluorimeter (Invitrogen, Eugene, OR) and 2100 Bioanalyzer (Agilent Technology, Santa Clara, CA).

Three 6-, 10-, and 13-kb SMRTbell libraries were prepared according to the PacBio sample preparation protocol (https://www.pacb.com/wp-content/uploads/2015/09/Guide-Pacific-Biosciences-Template-Preparation-and-Sequencing.pdf), including additional separation on a BluePippin system (Sage Science, Beverly, MA) and sequencing with C4-P6 chemistry, using 9 SMRT cells, as follows: 5 with non-size selected (6 and 10 kb) and 4 with size selected 13-kb libraries with a 360-minute collection time for each library. A total of 3,048 Gb sequencing data in 274,386 polymerase reads with mean subread lengths of 4,980 bp and *N*_50_ subread length of 7,635 bp were obtained (∼426× coverage) and *de novo* assembled using HGAP_Assembly.3 version 2.3.0 with default quality and read length parameters and 3 times polished using Quiver (7). The polished assemblies generated seven closed circular contigs (Table 1). The assembled sequences were annotated using the NCBI Prokaryotic Genomes Annotation Pipeline (PGAP) (8, 9). Previously, the *H*. *sinaiiensis* ATCC 33800 genome sequence was deposited in GenBank as 84 contigs based on short read sequencing as AOLR01000000.1.

**TABLE 1:** Summary of genome elements, DNA methyltransferase genes, and motifs identified in *Haloarcula sinaiiensis* ATCC 33800

Genetic element	Accession no.	Genome size (bp)	Genome coverage (×)	Methylase (RM^*a*^ system) name	Recognition motif^*b*^	Methylation RM type
Chromosome	CP073366	3,093,724	426.63	M.Hsi33800I	**C**TA**G**	II
				M.Hsi33800IV	CTC**A**(N6)**T**CG	I
Hsi_p540	CP073368	540,110	587.39	M.Hsi33800II	G**AT**C	II
				Hsi33800V	C**T**CC**A**G	II
Hsi_p388	CP073369	387,656	402.79			
Hsi_p204	CP073371	204,099	816.16			
Hsi_p139	CP073372	139,649	633.55			
Hsi_117	CP073367	116,596	568.55	M.Hsi33800III	C**A**G(N6)**T**CGC	I
Hsi_p55	CP073370	55,294	1,490.08			

a RM, restriction-modification.

b Modified bases and the bases opposite to them are in bold and underlined, respectively.

Four m6A and one m4C modified DNA motifs were detected by the SMRT motif and modification analysis version 2.3.0 (10–12). Scanning the genome using the Seqware program (13) predicted nine methyltransferase (MTase) genes in the genome. All five motifs and their corresponding MTase genes were predicted based on homology (14) to previously known MTases. The results are presented in Table 1 and have been deposited in REBASE (15).

### Data availability.

Sequences were deposited in the following NCBI databases under the indicated accession numbers: BioProject, PRJNA412908; BioSample, SAMN18811338; GenBank, CP073366 to CP073372; and SRA, SRS8750153.
